# Chronometric TMS-fMRI of personalized left dorsolateral prefrontal target reveals state-dependency of subgenual anterior cingulate cortex effects

**DOI:** 10.1038/s41380-024-02535-3

**Published:** 2024-03-26

**Authors:** Sarah Grosshagauer, Michael Woletz, Maria Vasileiadi, David Linhardt, Lena Nohava, Anna-Lisa Schuler, Christian Windischberger, Nolan Williams, Martin Tik

**Affiliations:** 1 Center for Medical Physics and Biomedical Engineering, Medical University of Vienna, Vienna, Austria; 2 Research Group Cognition and Plasticity, Max Planck Institute for Human Cognitive and Brain Sciences, Leipzig, Germany; 3 Department of Psychiatry and Behavioral Sciences, Stanford University, Stanford, CA USA

**Keywords:** Depression, Neuroscience

## Abstract

Transcranial magnetic stimulation (TMS) applied to a left dorsolateral prefrontal cortex (DLPFC) area with a specific connectivity profile to the subgenual anterior cingulate cortex (sgACC) has emerged as a highly effective non-invasive treatment option for depression. However, antidepressant outcomes demonstrate significant variability among therapy plans and individuals. One overlooked contributing factor is the individual brain state at the time of treatment. In this study we used interleaved TMS-fMRI to investigate the influence of brain state on acute TMS effects, both locally and remotely. TMS was performed during rest and during different phases of cognitive task processing. Twenty healthy participants were included in this study. In the first session, imaging data for TMS targeting were acquired, allowing for identification of individualized targets in the left DLPFC based on highest anti-correlation with the sgACC. The second session involved chronometric interleaved TMS-fMRI measurements, with 10 Hz triplets of TMS administered during rest and at distinct timings during an N-back task. Consistent with prior findings, interleaved TMS-fMRI revealed significant BOLD activation changes in the targeted network. The precise timing of TMS relative to the cognitive states during the task demonstrated distinct BOLD response in clinically relevant brain regions, including the sgACC. Employing a standardized timing approach for TMS using a task revealed more consistent modulation of the sgACC at the group level compared to stimulation during rest. In conclusion, our findings strongly suggest that acute local and remote effects of TMS are influenced by brain state during stimulation. This study establishes a basis for considering brain state as a significant factor in designing treatment protocols, possibly improving TMS treatment outcomes.

## Introduction

Transcranial magnetic stimulation (TMS) treatment of major depressive disorder (MDD) has recently gained importance with the FDA clearance of highly individualized therapy plans [1–5]. Multimodal investigations have shown that TMS modifies whole networks rather than only the stimulated area [6–10], providing the opportunity to interact with interconnected areas relevant for depression symptom relief via easily accessible targets, i.e. the left dorsolateral prefrontal cortex (DLPFC). This region exhibits strong negative functional connectivity (FC) to the subgenual anterior cingulate cortex (sgACC). Research has shown that the sgACC plays an important role in depression symptom severity and treatment response [11–15]. Analysis of early clinical studies revealed that therapeutic outcomes depend highly on the actual stimulation site within the left DLPFC and its FC to the sgACC [16, 17]. More recently, studies have emphasized the relevance of personalization based on individual anti-correlation patterns [18–22]. Although some treatment protocols lead to high remission and response rates [1, 2], studies still show high variability of TMS effectiveness in neurocognitive research and therapeutic applications [23–26]. Several factors may contribute to these variabilities, including definition of stimulation intensity and stimulation protocols [27] and target site [28]. Importantly, brain state is usually not regarded as a relevant parameter, although there is growing evidence that brain state can affect the TMS response [29–32]. Current treatment protocols perform stimulation during rest/unconstrained thoughts, i.e. the patients are asked to stay awake and to avoid motion [33]. In this setting, the underlying condition of the brain is largely ignored and the brain is treated as within a black box [34].

TMS needs to be interpreted as an interaction between TMS-induced current and ongoing neural processing (i.e. the brain state), as its neuronal impact significantly depends on the susceptibility of brain regions to stimulation [35–39]. This has been demonstrated in motor areas [40, 41], with the difference between active and resting motor threshold (rMT) [42] being probably the most intuitive example. Similar principles apply to other brain regions, as less activated neurons seem to show higher susceptibility to TMS and ceiling effects might prohibit additional activation of already highly activated neurons, resulting in inhibitory effects [35, 36, 43, 44].

To assess state-dependent effects, TMS can be applied during tasks in a chronometric fashion, i.e. at specific timepoints in relation to the task [45–51]. Utilizing the temporal precision of chronometric TMS and spatial accuracy of functional magnetic resonance imaging (fMRI), ongoing task processing can be influenced by TMS while the effects in terms of BOLD changes are recorded. Interleaved stimulation and imaging [52–54] allows for a comprehensive assessment of whole-brain TMS-induced BOLD changes and state-dependent effects beyond behavioral assessments. While this methodology has been applied mainly in cognitive research [48, 55–64], state dependency is also of high clinical relevance. Clinical studies in depression treatment found improved outcomes when standardizing brain state prior to or during stimulation [29–32]. In addition, a recent study [65] revealed the influence of EEG alpha phase during TMS pulses on modulations of sgACC FC. However, evidence for state-dependency of TMS effects in clinically relevant areas is still quite limited.

In the current study we applied chronometric interleaved TMS-fMRI to assess differences in TMS-induced BOLD response on a whole-brain level across different brain states. Emphasis was placed on the cognitive state of the brain. Specifically, we targeted nodes of the cognitive control network [66], which incorporates the DLPFC as well as the sgACC. Within this network, MDD manifests in terms of hypoactivation of the DLPFC co-occurring with hyperactivation of the sgACC [66]. Successful depression treatments, including TMS, have been found to normalize these two aspects by increasing DLPFC- and decreasing sgACC activity [3]. To assess the relevance of state-dependency for TMS targeting this network, we selected the N-back task [67–69] to induce standardized brain states. This decision was supported by the known interaction of the task with the cognitive control network and the decline in working memory performance associated with depression [70, 71].

Similar to a pioneering study investigating TMS during N-back task [62], we leveraged precise timing of pulses in relation to the task and performed chronometric TMS to an individualized resting-state connectivity informed DLPFC target. Consequently, we hypothesized that the neural response, as indicated by the BOLD-signal, would be dependent on the timing of TMS during the 2-back task. Specifically, we theorized that minor adjustments in TMS timing would result in distinct effects on the targeted brain regions, i.e. the DLPFC and sgACC. To account for non-specific effects we included TMS during 0-back as an active control condition.

## Materials and methods

### Participants

**Fig. 1:**
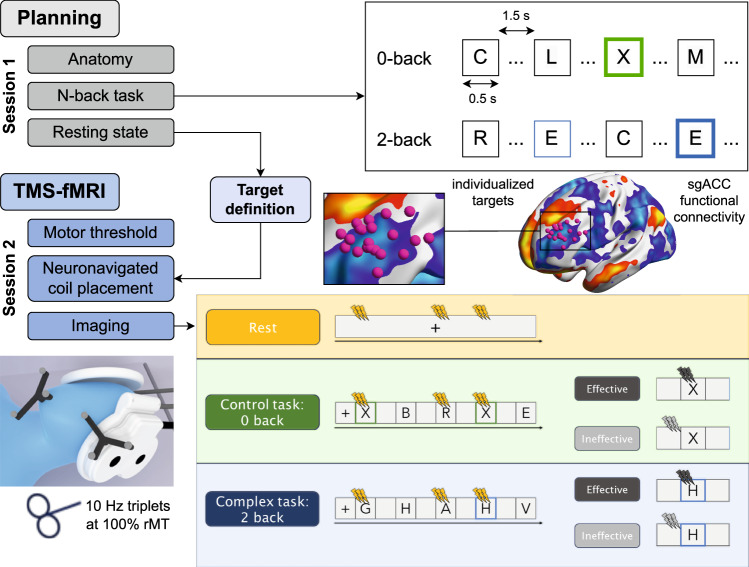
Overview of the conducted experiment, showing both measurement sessions and the task. The N-back task was performed at two difficulty levels (*N* = 0 and *N* = 2) in a blocked design, consisting of three blocks per difficulty. The 0-back condition was designed as an active control condition. During session 2, interleaved TMS bursts were delivered in 10 Hz triplets at 100% rMT to the individualized target site during three different states and considering two different timings for stimulation during the task.

26 healthy, young individuals were recruited through advertisements on the public boards for study recruitment in the General Hospital of Vienna as well as through university channels. Participants were carefully screened for contraindications to MRI/TMS, including implanted devices, claustrophobia, pregnancy as well as history of epilepsy, neurological or psychiatric diseases and severe chronic illnesses. All participants provided written informed consent. The study was approved by the local ethics committee of the Medical University of Vienna and in accordance with the Declaration of Helsinki. Two participants were excluded after session 1, as the TMS intensity required to detect a reliable motor response could not be achieved. Four participants were excluded due to motion, resulting in 20 participants (12 female, 8 male, age range 18–39, mean ± std 25 ± 6 y) for the final analysis. Measurements were performed in two sessions, separated by a minimum of two days. The workflow is shown in Fig. 1. Imaging data was acquired on a Siemens PrismaFit 3 T MR scanner (Siemens Healthineers, Erlangen, Germany). In the first session, imaging data required for TMS-fMRI planning was acquired and participants were familiarized with the task. Based on the acquired resting-state data, the individual resting-state networks of the sgACC were calculated and used for target definition [20]. In the second session, interleaved TMS-fMRI was performed during rest and task paradigms.

### Image acquisition

In the first session, the manufacturer’s 64-channel head coil was used for resting-state acquisition (TE = 38 ms, TR = 2000 ms, flip angle 77°, voxel size 1.8 × 1.8 × 2 mm³, MB = 4), a run of the N-back task (TE = 38 ms, TR = 1000 ms, flip angle 60°, voxel size 3 × 3 × 3 mm³, 40 slices, 0.6 mm slice gap, MB = 4) and T1-weighted imaging (TR = 2100 ms, TE = 3.67 ms, flip angle 8°, voxel size 1 mm isotropic). The resting-state acquisition paradigm was selected due to previously evaluated reliability of sgACC-DLPFC FC networks [72].

During the second session, interleaved TMS-fMRI was performed using three dedicated 7-channel receive arrays [73, 74]: one mounted directly underneath the TMS coil placed on the left DLPFC, the second on the contralateral DLPFC and a third underneath the participant’s head (occipital position) to achieve whole-brain coverage. Functional imaging was performed with the same parameters as the N-back acquisition during session 1, enabling the interleaving of TMS bursts with image acquisition. In addition, an anatomical MP2RAGE scan (TE = 2.98 ms, TR = 4000 ms, TI1 = 700 ms, TI2 = 2500 ms, flip angle 4°/5°, voxel size 1 mm isotropic, 160 slices) was acquired. Python-based software was developed for synchronizing tasks, image acquisition, and TMS pulses. MR triggers and task-based triggers were fed into an analog in-house built AND-gate, which forwarded triggers at the correct time points to the stimulator. To avoid interactions of TMS pulses with image acquisition, an additional delay of 80 ms for triggering was defined at the stimulator.

### Neuronavigation

To achieve individualized targets for each participant, the resting-state data acquired during the first session was analyzed. Preprocessing of data was performed using a dedicated in-house developed pipeline (RSTools, for details see Supplementary Material). To determine the TMS target network, seed voxel based correlation analysis [75] was used. The seed was defined as a 10 mm radius sphere representing the sgACC centered at MNI 6, 16, −10 and masked for gray matter [16]. For target definition and neuronavigation the Brainsight software suite (Rogue Research Inc., Canada) was used. The coil position was defined by masking the participant’s FC map with a left DLPFC mask as defined by Cash et al. [20] and projecting it onto the extracted brain surface. The most negative cluster on the surface was identified and defined as the coil center. Coil orientation was defined with a 45° angle between coil handle and brain midline.

TMS was performed using a MagProX100 stimulator and the MR-compatible MRi-B91 (MagVenture, Farum, Denmark) TMS coil. During session 2, the stimulation intensity was defined for each participant based on resting motor threshold (rMT). rMT was determined as the lowest stimulator output resulting in 5 out of 10 visible twitches of the right first dorsal interosseous muscle [76]. Motor threshold definition was performed inside the scanner room, using the sandwiched TMS and 7-channel receive coil array.

Using a Polaris neuronavigation camera (NDI, Waterloo, Canada) and 3D-printed metal-free optical trackers for participant (mounted onto a mouthpiece) and TMS coil, the coil was positioned on the individual DLPFC target and fixed using a suitable holder (MagVenture, Farum, Denmark).

### Task and stimulation paradigm

The N-back task [67, 68] consisted of a 30 s baseline at the beginning, six task blocks, and 30 s baseline between blocks. Each task block (0-back, 2-back) was repeated three times in a randomized order. The 0-back condition and the different stimulation onsets were designed as active controls to account for sensory-motor responses, TMS-related attention effects and task specific effects of no interest (visual processing including visual attention). During 0-back, participants were asked to indicate the appearance of the letter “X” by button press. In the 2-back condition, participants were instructed to press a button if the currently displayed letter matched the letter presented two positions earlier (“matching” of stimuli [77]). This required participants to continuously update and recall information, making the 2-back task more challenging and requiring working memory capacity. Each block consisted of 40 individual letters, 8 of them being target letters, i.e. requiring a button press response. Letters were displayed for 0.5 s, followed by a blank of 1.5 s (see Fig. 1).

During interleaved TMS-fMRI, TMS was applied between slice acquisitions in triplets of 10 Hz at 100% of rMT. Each task was combined with three conditions for TMS: (1) no stimulation, (2) stimulation 250 ms before letter appearance, and (3) stimulation 150 ms after letter appearance. The latter was hypothesized to interfere effectively with the participant’s matching process [74] (i.e. the cognitive state of interest), as well as present a more susceptible state of the DLPFC to TMS. From here on, we refer to stimulation after letter onset as “effective timing”. Stimulation prior to letter onset was hypothesized to not interact directly with matching processes, as no visual processing did take place yet and will from here on be referred to as “ineffective timing”. Stimulation was performed on target letters as well as on an equal number of random non-target letters for both difficulties (16 TMS triplets per condition). Each task/TMS combination was delivered in a block-wise fashion, matching the total number of six blocks. The order of blocks was pseudo-randomized for each participant, with two blocks of no stimulation in the middle of the run. In addition to stimulation during the task, a run of stimulation during rest was performed. This run was identical in timing and stimulation pattern to a task-TMS run, however, the participant was instructed to fixate a cross and think about nothing in particular. The order of rest and task runs was counterbalanced between participants.

### Behavioral analysis

To evaluate the potential behavioral effects of TMS under different task conditions and stimulation timings, we calculated mean reaction time (RT) for correct trials and accuracy for each participant. Additionally, we computed the balanced integration score (BIS), as described by [78]. This score combines RT and accuracy into one measure, giving them equal weights by z-standardization. Positive BIS indicates above average performance for a given task, while negative BIS corresponds to below average performance. Z-standardization was based on the average performance metrics for a specific task (0-back, 2-back), independent of TMS condition (no TMS, effective/ineffective timing). Paired t-tests between the behavioral parameters obtained during blocks without and with TMS were performed at *p* < 0.05 to assess whether TMS affected behavior. For the BOLD effects of interest effect size was calculated in terms of Cohen’s d. Due to button box issues that prevented response logging for one participant, we excluded this individual from the behavioral analysis. The behavioral data analysis thus includes data from 19 participants.

### Preprocessing of TMS-fMRI data

Acquired EPIs were preprocessed including ANTS [79] N4BiasFieldCorrection and despiking (AFNI) as well as SPM12 realignment, normalization to MNI space and smoothing with a Gaussian kernel of 6 mm full width at half maximum. Image sets were visually checked for quality as well as based on framewise displacement (FD, [80]). Severe motion artifacts, corresponding to max. FD > 1.5 mm or mean FD > 0.15 mm caused the exclusion of 4 datasets.

### Statistical analysis

Analysis of preprocessed TMS-fMRI data was conducted using SPM12. Single-subject first-level analysis was performed using a general linear model. Linear regression was performed for each voxel based on generalized least squares with a global approximate AR(1) autoregression model.

For TMS during rest, one participant had to be excluded as no stimulation time point information was available due to corrupted log-files. For GLM analysis, individual stimulation timepoints were modeled as events. In the N-back runs, task blocks and button-press events were modeled with regressors to account for all activation changes associated with the task itself (0-back, 2-back) and motor response. For blocks with stimulation, individual TMS pulses were additionally included as separate events, differentiating between the different states as described previously. This resulted in a total of seven regressors: 0-back task, 2-back task, button press, 0-back effective TMS, 0-back ineffective TMS, 2-back effective TMS, 2-back ineffective TMS. In addition, six motion parameters derived from image realignment were included. 2-back TMS estimates were contrasted against the corresponding 0-back TMS responses, i.e. TMS with equivalent timing, to account for non-specific TMS effects. Same analysis was performed for data acquired without TMS during a separate session, by modeling artificial events identical to TMS timing (i.e. events which mimic stimulation timepoints, although no stimulation occurred). To assess whether the event timing causes changes in modeled brain response, the same analysis was performed for data acquired without TMS during a separate session, by modeling artificial events identical to TMS timing. These results were subsequently compared to results for interleaved TMS-fMRI.

Second-level analysis was performed to address the research question if TMS during 2-back at the correct time point for cognitive processing results in distinct local and remote BOLD changes. The results were double controlled for cognitive state (i.e. effective and ineffective timing) and task processing (i.e. 2-back versus 0-back). Specifically, a model was created to investigate differences between timings in the 2-back task, using TMS during the 0-back task as an active control condition. Subject-level contrasts were further analyzed on a group level in a flexible factorial model. Both timings were contrasted against each other, resulting in the target contrast (2-back effective > 0-back effective) vs (2-back ineffective > 0-back ineffective). Second, group level t-tests were performed for TMSin all states to assess TMS BOLD response considering inter-subject variance. Within this second analysis, results were not controlled for unspecific task or TMS-related effects.

Significance levels for all group-level image analysis were set to *p* < 0.05 for cluster-level family-wise error (FWEc) correction (with *p* < 0.001 for cluster definition). To investigate the effect of TMS during different states on the targeted sgACC, region of interest (ROI) analysis of percent signal change (PSC) was performed using Python. The ROI was defined a-priori as a sphere with 10 mm radius (MNI 6, 16, −10 [16]). In addition, a more pregenual area of the ACC (pgACC) which has been associated with increased glucose metabolism [81] as well as low frequency fluctuations in MDD [82], was investigated (sphere at MNI 0, 42, 6). For each participant, individual electric field hotspots were calculated post-hoc based on SimNIBS [83, 84] simulations of the actual coil position and orientation (see Supplementary Fig. S1). These simulations were used to derive a left DLPFC group ROI based on the average electric field hotspot center of mass (see Supplementary Material, sphere at MNI −43, 39, 27). Finally, personalized ROIs were placed on each individual’s hotspot. Extracted mean PSCs for each ROI were tested for statistical significance in one sample as well as paired *t*-tests.

## Results

### TMS targets and stimulation during rest

**Fig. 2:**
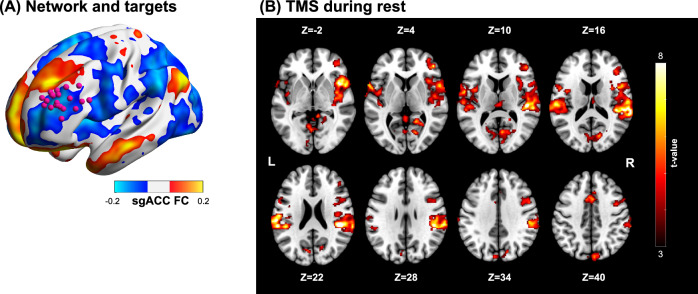
Individualized targeting and TMS during rest. Group-averaged resting-state functional connectivity for the sgACC seed and individual functional connectivity guided target positions are shown in (**A**). **B** displays *t*-value group maps of TMS during rest at *p* < 0.05 FWE corrected at cluster level (*p* < 0.001 cluster defining threshold).

Coil positions were derived based on individual FC to the sgACC - individual targets are shown in Fig. 2A, overlaying an average FC map of the sgACC. rMT ranged between 63 and 95% of the maximum stimulator output, corresponding to dI/dt of 106–178 A/µs. Resulting E-field magnitude hotspots (99.9-th percentile) corresponded to values between 65 and 118 V/m. Figure 2B shows the activation map for TMS during rest. Statistically significant clusters (*p* < 0.001 cluster detection, *p* < 0.05 FWEc) are listed in Table 1. Besides sensory and auditory activations in the temporal superior lobes as well as insulae, significant activations can be found in the right DLPFC, thalamic areas, mid cingulate cortex, SMA and in the cerebellum.

**Table 1:** Statistically significant (*p* < 0.05, FWE corrected on cluster level, *p* < 0.001 cluster defining threshold) clusters for TMS during rest and TMS during different cognitive states.

*State*	Cluster			Peak			MNI	Region
	k_E_	*p*_FWE-corr_	*p*_unc_	*p*_FWE-corr_	T	*p*_unc_		
Rest	5942	0.000	0.000	0.000	14.53	0.000	44 −32 22	Rolandic_Oper_R
	2461	0.000	0.000	0.003	9.38	0.000	−60 −24 22	SupraMarginal_L
	750	0.000	0.000	0.004	9.17	0.000	8 −4 60	Supp_Motor_Area_L
	104	0.026	0.001	0.041	7.80	0.000	−34 −52 −24	Cerebelum_6_L
	501	0.000	0.000	0.072	7.50	0.000	46 40 6	Frontal_Inf_Tri_R
	1261	0.000	0.000	0.199	6.90	0.000	14 −74 18	Calcarine_R
	178	0.000	0.000	0.275	6.66	0.000	16 −52 2	Lingual_R
	383	0.000	0.000	0.362	6.44	0.000	−6 −74 −20	Cerebelum_6_L
	161	0.002	0.000	0.846	5.52	0.000	8 −42 −2	Lingual_R
	121	0.012	0.000	0.898	5.40	0.000	2 −30 84	Paracentral_Lobule_R
	127	0.009	0.000	0.910	5.36	0.000	2 −20 12	Thal_PuM_R
0-back effective	4741	0.000	0.000	0.000	11.56	0.000	46 −41 24	SupraMarginal_R
	2775	0.000	0.000	0.021	7.98	0.000	−44 −26 12	Temporal_Sup_L
	123	0.013	0.000	0.367	6.26	0.000	12 −58 −10	Cerebelum_4_5_R
	111	0.022	0.001	0.527	5.96	0.000	−12 −62 −26	Cerebelum_6_L
	240	0.000	0.000	0.877	5.33	0.000	54 0 48	Precentral_R
	265	0.000	0.000	0.884	5.31	0.000	10 −4 66	Supp_Motor_Area_R
	109	0.024	0.001	1.000	4.47	0.000	0 −54 62	Precuneus_L
0-back ineffective	2898	0.000	0.000	0.011	8.30	0.000	46 −32 18	Rolandic_Oper_R
	1282	0.000	0.000	0.075	7.29	0.000	−46 −38 28	SupraMarginal_L
	148	0.009	0.000	0.857	5.30	0.000	10 −62 −14	Cerebelum_6_R
	105	0.045	0.002	0.995	4.69	0.000	6 −4 68	Supp_Motor_Area_R
2-back effective	4352	0.000	0.000	0.000	10.94	0.000	46 −30 20	Rolandic_Oper_R
	2919	0.000	0.000	0.006	8.63	0.000	−54 −34 18	Temporal_Sup_L
	202	0.001	0.000	0.122	7.05	0.000	−46 20 26	Frontal_Inf_Tri_L
	118	0.015	0.000	0.929	5.20	0.000	46 26 14	Frontal_Inf_Tri_R
2-back ineffective	3752	0.000	0.000	0.000	12.75	0.000	48 −28 26	SupraMarginal_R
	705	0.000	0.000	0.042	7.60	0.000	−58 −22 18	SupraMarginal_L
	101	0.024	0.001	0.048	7.53	0.000	−2 −66 −16	Vermis_6
	131	0.006	0.000	0.071	7.33	0.000	34 10 12	Insula_R
	245	0.000	0.000	0.465	6.12	0.000	−54 0 6	Rolandic_Oper_L
	107	0.018	0.000	0.796	5.55	0.000	44 2 54	Frontal_Mid_2_R

For each cluster the peak *t*-value is displayed. Anatomical regions were assigned using AAL3 toolbox [102] and local maxima labeling.

### Brain response during chronometric TMS

**Fig. 3:**
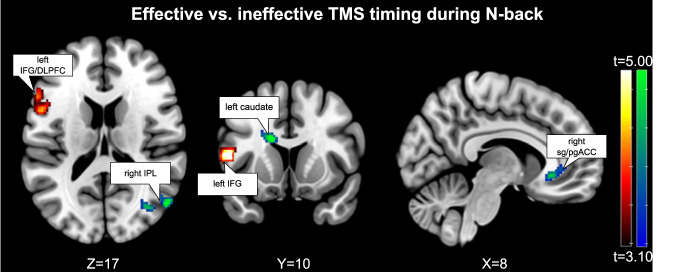
Brain regions showing increased (hot) and decreased (blue-green) activation for TMS effective vs. ineffective timing during N-BACK (thresholded at *p* < 0.05, family-wise error corrected on cluster level). Compared to TMS during 0-back as a control condition, effective vs. ineffective stimulation during 2-back leads to modulation of key regions linked to clinical TMS effects. Importantly, areas of the left DLPFC/inferior frontal gyrus (IFG) show higher activation and subgenual anterior cingulate cortex (sgACC), right inferior parietal lobule (IPL) and striatal areas lower activation if stimulation was performed in the effective timing.

Analyzing TMS-timing effects on the N-back target contrast (2-back actively controlled by 0-back) showed that effective timing of TMS (post-letter onset) was associated with higher activation in left inferior frontal gyrus (IFG) extending to areas of the left DLPFC on a group level (Fig. 3 and Table 2). Importantly, decreased activation with effective compared to ineffective timing can be found close to the subgenual ACC regions (peak at MNI = 2, 30, −6; *t* = 4.82) as well as in caudate regions. The contrast for our main result in sgACC revealed a Cohen’s d of 0.61, which can be defined as a large effect and a subsequent estimated post-hoc power of 0.84.

**Table 2:** Brain regions showing statistically significant (*p* < 0.05, FWE corrected on cluster level, *p* < 0.001 cluster defining threshold) increased and decreased activation for TMS during effective timing compared to ineffective timing.

	Cluster			Peak			MNI	Region
	k_E_	*p*_FWE-corr_	*p*_unc_	*p*_FWE-corr_	T	*p*_unc_		
Effective > ineffective	353	0.000	0.000	0.016	5.67	0.000	−50 12 10	Frontal_Inf_Oper_L
				0.241	4.84	0.000	−44 28 6	Frontal_Inf_Tri_L
				0.878	4.18	0.000	−54 20 16	Frontal_Inf_Tri_L
Ineffective > effective	309	0.000	0.000	0.019	5.62	0.000	−14 12 24	Caudate_L
				0.063	5.27	0.000	−18 2 30	Caudate_L
				0.497	4.55	0.000	−12 −12 30	Cingulate_Mid_L
	244	0.001	0.000	0.076	5.22	0.000	52 −68 16	Temporal_Mid_R
				0.117	5.08	0.000	40 −70 20	Temporal_Mid_R
				0.758	4.31	0.000	50 −68 32	Angular_R
	122	0.046	0.002	0.252	4.82	0.000	2 30 −6	ACC_sub_R
				0.574	4.48	0.000	12 26 −4	Caudate_R
				0.999	3.75	0.000	8 36 0	ACC_sub_R

The target contrast was (2-back effective > 0-back effective) vs. (2-back ineffective > 0-back ineffective). For each cluster the three highest *t*-value peaks are displayed. Regions were assigned using AAL3 Toolbox [102] and local maxima labeling.

**Fig. 4:**
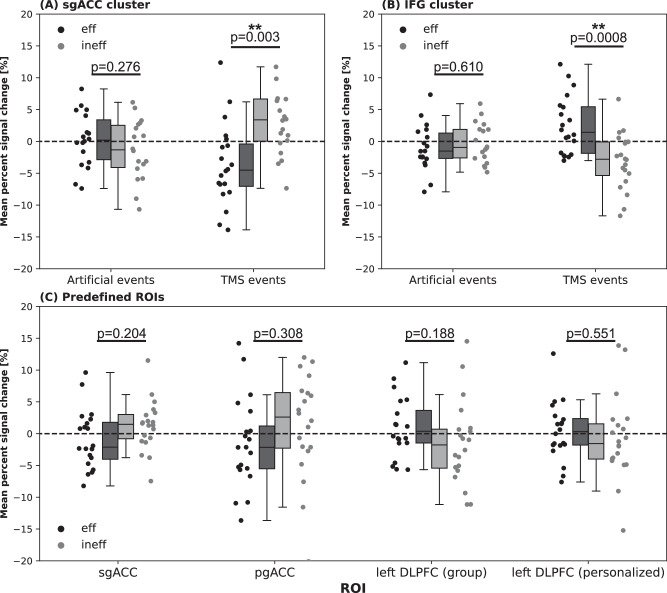
ROI analysis for effective and ineffective timing during N-back task. For each ROI, subject-level mean PSCs for the effective (2-back effective > 0-back effective) and ineffective (2-back ineffective > 0-back ineffective) condition were extracted. (**A**) and (**B**) show ROI analysis of clusters showing significant differences in activation for differential timing of TMS in comparison to artificial events, i.e. same timepoints of the task but no TMS was performed. In (**C**) extracted mean PSCs for the predefined ROIs during TMS with different timings is displayed. *P* values correspond to paired *t*-tests between timings.

Figure 4A, B shows the comparison of PSC of the detected clusters (sgACC, IFG) with pure task effects as derived from the artificial events analysis. Statistically significant pairwise difference (*p* < 0.05) between timings could only be detected for TMS, but not for the artificial events, i.e. if no TMS was performed. In the predefined ROIs no statistically significant difference between effective and ineffective TMS timings could be detected in a paired t-test (Fig. 4C).

### Brain response in relevant ROIs during rest and task-based stimulation

Statistically significant clusters are shown in Table 2. On a group-level stimulation during rest did not result in significant effects in the sgACC (mean ± standard deviation: −0.260 ± 1.760, one sample t-test: *p* = 0.537, t = −0.629). Negative mean PSCs could be detected in all task-based states (0-back effective: −1.02 ± 3.89, p = 0.267, t = −1.144; 0-back ineffective: −2.07 ± 2.71, *p* = 0.003, *t* = −3.334; 2-back effective: −1.91 ± 3.81, *p* = 0.042, *t* = −2.181; 2-back ineffective: −0.83 ± 2.89, *p* = 0.229, *t* = −1.243), with statistical significance reached for 0-back ineffective and 2-back effective timing.

In the pgACC a statistically significant effect was detected for TMS during rest (p = 0.009, t = −2.896) and 2-back effective timing (*p* = 0.03, *t* = −2.351). In the left DLPFC group-ROI a significant positive effect was detected for 2-back effective timing (*p* = 0.005, *t* = 3.189), while personalized ROIs showed significant effects during rest (*p* = 0.002, *t* = 3.686). Unthresholded activation maps for TMS during different states (rest, 0-back effective, 0-back ineffective, 2-back effective, 2-back ineffective) as well as ROI analysis can be found in the Supplementary Material.

### Behavioral results

Mean accuracy across both tasks and TMS conditions was notable high (0-back: 1 ± 0, 0-back effective TMS: 0.983 ± 0.051, 0-back ineffective TMS: 0.991 ± 0.034, 2-back: 0.974 ± 0.038, 2-back effective TMS: 0.961 ± 0.031, 2-back ineffective TMS: 0.942 ± 0.066). Accuracy was lower in the 2-back condition if TMS was performed with ineffective timing (*p* = 0.043), but not during any other condition.

For 0-back without TMS the mean RT was 0.471 ± 0.064 s, with effective TMS 0.480 ± 0.111 s and for ineffective TMS 0.436 ± 0.050 s. In comparison, mean RT for 2-back without TMS was 0.631 ± 0.166, with effective TMS timing 0.564 ± 0.138 s and with ineffective timing 0.588 ± 0.224 s. There was a significant decrease in RT for the 0-back task with ineffective TMS (*p* = 0.003) compared to without TMS, indicating faster responses with TMS. Effective timing did not result in a significant change in RT (*p* = 0.727). For the 2-back task RT was not statistically significantly different from without TMS(effective: *p* = 0.081; ineffective: *p* = 0.379). BIS scores did not indicate significant changes in overall task performance. All behavioral parameters can be found in the Supplementary Material.

## Discussion

Within this study we investigated state-dependency by applying TMS interleaved with fMRI during rest and in a chronometric fashion during a task. Emphasis was put on the left DLPFC-sgACC axis as part of the cognitive control network, pertinent to TMS depression treatment. Importantly, we examined how timing of TMS relative to cognitive task processing influences target engagement. Based on the engagement of the DLPFC and the known interactions with depression, a 2-back memory task was chosen. To account for attention-related and non-specific TMS effects we included a 0-back active control condition. To our knowledge, this study is the first application of chronometric interleaved TMS-fMRI. Combining the spatial accuracy of fMRI with the temporal precision of TMS, this methodology offers a unique perspective on state-dependent effects.

### TMS during rest

In general, TMS response during rest co-localizes with prior investigations [85, 86]. However, we present novel interleaved TMS-fMRI data for personalized targets in the left DLPFC. Notably, we observed sub-threshold group-level activation at the stimulation site, adding to the discussion about activation beneath the coil [87]. The use of 100% rMT in our study, as opposed to higher intensities in previous work, may have influenced these outcomes. In addition, variance in actual target sites across participants could contribute to reduced group-level effects. On a single-subject level only some participants showed increased BOLD responses under the coil, while others did not. Interestingly, ROI analysis revealed statistically significant PSC in personalized DLPFC targets, i.e. in subject-specific areas of high E-field. This highlights the importance of verification of actual stimulation sites as well as E-field modeling, as areas of high E-field are not necessarily identical to the coil center (see Supplementary fig. S7). In addition, there might be an effect of individual participants’ state fluctuations that interact differently with stimulation in the absence of a task [65].

### TMS timing

For stimulation during task, we selected two timings which participants were not able to differentiate between. During 2-back, individual processes of working memory (encoding, maintenance, retrieval) happen simultaneously, i.e. cannot be separated in the temporal domain [77, 88]. Nevertheless, it has been shown previously that in the timeframe which was associated with effective stimulation (approx. 100 ms after letter onset), an increase in frontal theta power occurs [89]. Interestingly, modulation of this parameter prior to rTMS treatments has also been associated with improved clinical response [30]. Furthermore, we expected a more optimal pre-engagement of the targeted network during this time point, resulting in higher susceptibility of the DLPFC to TMS. In contrast, ineffective timing refers to the stimulation occurring within blanks shortly before the appearance of the next letter, i.e. no new stimulus had to be processed and the brain is in a maintenance/attention phase. Thus, we expected less pre-engagement of the DLPFC.

### Behavioral effects

Previous studies [50, 90–93] indicate that priming of participants with TMS before stimuli onset may improve cognitive function. This also partly matches with results of this study, as statistically significant improvements in reaction time could be identified for TMS pulses applied pre letter onset (ineffective timing) compared to no TMS during 0-back task (see Supplementary fig. S2). For the effective timing we could not detect significant changes in behavior, which again indicates that depending on the timing in relation to the task, based on the temporal activation pattern of different network nodes, TMS results in differential behavioral effects. For the 2-back task only, TMS with ineffective timing resulted in a statistically significant decrease in accuracy, while no other parameter was affected. This finding contrasts with prior research, where TMS was shown to have beneficial effects on working memory performance [59, 89]. Specifically, studies [59] and [89] reported enhanced working memory capabilities following TMS intervention, suggesting a potential modulatory role of TMS in cognitive tasks. One plausible explanation could be that participants in our sample achieved almost 100% of accuracy in the baseline measurements. Accuracy, however, is based on a total number of 40 trials per condition, with only 8 true positives and 32 true negatives. Thus, effects of missing a correct trial on accuracy are comparably small. Considering the young age of our study population administering the 2-back condition of the n-back task might have been relatively easy for our participants which might also explain low behavioral effects of TMS. The interaction between TMS effects, individual’s ability and task difficulty has been shown previously [44, 58, 94]. The results of the current study might therefore inform future studies adjusting task difficulty individually [39]. Additionally, we did not observe consistent changes (either in positive or negative direction) in reaction time at the group level. This could be associated with the specific timing of stimulation in relation to the task, i.e. TMS was probably not performed during ideal time windows to enhance reaction time. These ideal time windows might also differ between participants. However, affecting cognitive function was not the aim of this study and would also require another TMS target [63].

### Chronometric TMS during the 2-back task

We identified the changes in BOLD effects solely related to different timings of TMS relative to cognitive processing in a 2-back condition by contrasting for non-specific TMS as well as non-specific task effects on a single-subject level. While effective (post-letter onset) timing of TMS revealed higher activations compared to ineffective (prior to letter onset) timing in the left DLPFC/IFG, decreased activation could be found in areas of the striatum, the right IPL and areas of the sgACC. Left IFG is known to be activated during verbal N-back [68], representing a task relevant area which shows increased activity with post-letter onset TMS timing even though the stimulation target was located more anterior. As no statistically significant changes in behavioral data could be found with TMS for the 2-back task, this might indicate a compensatory mechanism of action to counteract the distraction caused by the TMS burst [39, 95, 96]. This corroborates former findings by Webler et al. [62], who found a similar hotspot when comparing only 2-back TMS vs. 0-back TMS. However, during their study, stimulation was performed at unsynchronized timepoints in relation to the task, presenting an average response over all task phases.

Areas showing decreased activation with effective timing comprise among others striatal areas and areas of the ACC. This modulation of TMS effects indicates that timing and underlying state could play an important role for the effectiveness of clinical TMS, as these areas are considered highly relevant in depression treatment. Using TMS-fMRI, the general possibility of modulating activity within these areas has been shown previously [9, 86, 97]. Here, we show that chronometric TMS differentially affects these areas, independent of non-specific effects.

### State dependency of acute TMS effects

We identified that DLPFC-TMS effects on the sgACC highly depend on the underlying brain state. During rest, variability in terms of effect direction within the pool of participants resulted in no significant effect on a group level on the sgACC. Across participants, mean sgACC PSCs were statistically significant below zero only for TMS during 0-back with ineffective timing and for TMS during 2-back with effective timing. TMS during these states resulted in more consistent sgACC effects between participants, indicating the potential to standardize TMS response across participants by stimulating during a clearly defined optimal state. In addition, TMS response below the coil, i.e. on the left DLPFC, critically depends on the underlying state. Interestingly, statistically significant activation of personalized DLPFC ROIs could be detected for TMS during rest. Thus, task as well as timing of TMS in relation to the task is essential, as the state of the stimulation site and the underlying network highly depends on the phase of the task and thus also the susceptibility to TMS is affected [39, 43].

### Clinical relevance

Based on investigations in healthy participants we show that chronometric TMS is feasible and differentially modulates clinically relevant regions during task. The stimulation protocol emulates parameters of an actual TMS treatment, in terms of frequency and FC-derived individual target. Previous pioneering work [65] on TMS state dependency using EEG phases showed similar effects in both, depressive and healthy cohorts, but even higher effect magnitude in depressed individuals. Furthermore, it has been suggested that psychotherapy adjacent to rTMS may enhance treatment response[31, 32]. Similar effects might occur for task-induced state dependency, however, this still needs to be determined. In our study we could reveal that slight differences in timing differentially engage the targeted areas within the cognitive control network. Importantly, we could achieve higher activation in the IFG and decreased response in the sgACC with effective timing compared to ineffective timing. This is the same direction of effects required for counter-acting dysregulated activations within the targeted network during depression. Furthermore, we could achieve negative sgACC responses during state-based stimulation in a larger number of participants compared to during rest. These findings are particularly relevant as the sgACC’s BOLD response to TMS has recently been linked to the antidepressant effects of rTMS treatments [98]. Our results suggest that state-dependent stimulation can enhance acute sgACC effects through optimal pre-engagement of the target network, which could also further improve efficacy of TMS treatments [29–31]. However, stimulating during the wrong state could also render stimulation ineffective. Thus, further research is needed to identify the optimal state for TMS depression treatment prior to considering the inclusion of brain state into treatment protocols. Recent developments indicate that highly intense treatment paradigms including a large number of pulses in a relatively short amount of time resulted in comparable or superior outcomes compared to previous studies [1, 2, 4]. Based on the results derived from our study, one contributor to the improved response rates could be that by stimulating during different time points throughout a day, the probability of hitting a preferable state is increased and could also compensate for stimulations during suboptimal timepoints.

### Limitations

This study is limited by the small sample size of healthy participants only. Further validations in larger as well as clinical populations are needed. The targeted network as well as activation patterns during our task conditions overlap with the cingulo-opercular network, as defined in [99], which is also activated during uncomfortable stimuli [100]. However, we could also detect overlaps with the resting-state networks which have previously been associated with changes in functional connectivity after TMS [86] (Supplementary fig. S8). It is challenging to differentiate between effects caused by actual network stimulation and non-specific TMS effects. Considering that sham TMS may not be sufficient as a control paradigm [101], we think that including an active control condition is the best possible option within this study design. Importantly, this study overcomes sham effects by comparing different TMS timings during different tasks while stimulation protocol and target remain constant. Stimulation with slightly different timings as well as during different tasks acted as active control conditions. All TMS conditions create identical somatosensory and auditory effects, thus, differences in brain response are associated with the interaction of TMS with the underlying brain state. Finally, it needs to be mentioned that, although we regressed out general task activation, timing effects might be partly associated with time-dependent task activation. However, analysis of task data based on artificial events did not confirm this.

### Conclusions

In conclusion, the current study could show that underlying brain state affects acute TMS effects in the targeted sgACC. As current research suggests that clinical response is associated with direct sgACC BOLD effects, more consistent/stronger engagement of deep target areas could lead to increased treatment successes. By incorporating these insights on state dependency of the DLPFC-sgACC axis into treatment protocols, e.g. by adjusting frequency and duration of TMS sessions or combining stimulation with tasks, there is a potential to enhance TMS responses. Our findings pave the way for a more nuanced approach to TMS treatment, where both the location and the timing of stimulation are tailored to maximize target engagement. As the field of neuromodulation continues to evolve, such insights could be key to enhancing treatment response and consistency of TMS outcomes.

## Supplementary information

Supplementary Material

## Data Availability

All anonymized data and analysis codes are available upon request in accordance with the requirements of the institute, the funding body, and the institutional ethics board.

## Code availability

All anonymized data and analysis codes are available upon request in accordance with the requirements of the institute, the funding body, and the institutional ethics board.
