# Supplementary material for: Chronometric TMS-fMRI of personalized left dorsolateral prefrontal target reveals state-dependency of subgenual anterior cingulate cortex effects
Source: Mol Psychiatry. 2024 Mar 26;29(9):2678–88. doi: 10.1038/s41380-024-02535-3 (PMC11420068; doi:10.1038/s41380-024-02535-3)
Supplement: Supplementary file 1 — Supplementary Material [file 41380_2024_2535_MOESM1_ESM.docx]

**Chronometric TMS-fMRI of personalized left dorsolateral prefrontal target reveals state-dependency of subgenual anterior cingulate cortex effects**

***Supplementary information***

**Authors:** Sarah Grosshagauer ^1^, Michael Woletz ^1^, Maria Vasileiadi ^1^, David Linhardt ^1^, Lena Nohava ^1^, Anna-Lisa Schuler ^2^, Christian Windischberger ^1^, Nolan Williams ^3^, Martin Tik ^1,3*^

**Affiliations:**

1. Center for Medical Physics and Biomedical Engineering, Medical University of Vienna, Austria
2. Max Planck Institute for Human Cognitive and Brain Sciences, Leipzig, Germany
3. Department of Psychiatry and Behavioral Sciences, Stanford University, Stanford, USA

***Supplementary methods***

**Interleaved TMS-fMRI.** The EPI sequence was defined in a way that scanner triggers were sent out with each volume. MR scanner triggers were subsequently forwarded to a control PC, which started the task and provided triggers for the TMS pulses. For synchronizing the task with image acquisition and TMS pulses, an in-house developed Python software based on parallel port extension (PyParallel), QTimer and the Twisted framework was used. Trigger windows for TMS pulses based on the task were forwarded from the control PC to an analogue AND-gate. Second input to the gate was the MR scanner trigger signal. The output of the gate was directly connected to the TMS stimulator, i.e. when MR volume trigger and task trigger matched, the signal was forwarded without further delay to the stimulator. To account for potential interactions with image acquisition, an additional delay of 80 ms for triggering was defined at the stimulator. This delay ensured a time difference between slab excitation (MR scanner trigger) and TMS pulse. For differential timing of TMS in relation to the task (effective vs. ineffective timing), the task paradigm was slightly shifted in relation to the scanner triggers.

**Electric field modeling.** The TMS coil was placed on the individualized targets using neuronavigation and 3D printed lightweight trackers. The placement of the coil was saved before the participant entered the scanner bore and checked after measurements to make sure that no significant coil displacement occurred during the measurement. Based on these recorded coil position and orientation, electric field modeling was performed using SimNIBS 4.0.0 [1, 2] for each participant. Thus, tissue segmentation of the high resolution anatomical images acquired during session 1 was performed with the charm algorithm [3]. All electric fields were subsequently normalized to MNI space. Electric field hotspots were calculated using Python by detecting the 99.9-th whole brain percentile, binarizing it and determining the center of mass for this ROI using the center-of-mass function implemented in scipy.
(<https://docs.scipy.org/doc/scipy-0.14.0/reference/generated/scipy.ndimage.measurements.center_of_mass.html>, figure S1). The determined E-field hotspot was subsequently used for personalized ROI analysis in the left DLPFC. In addition, we calculated the average center of mass across participants to get a representative group-level ROI.

**
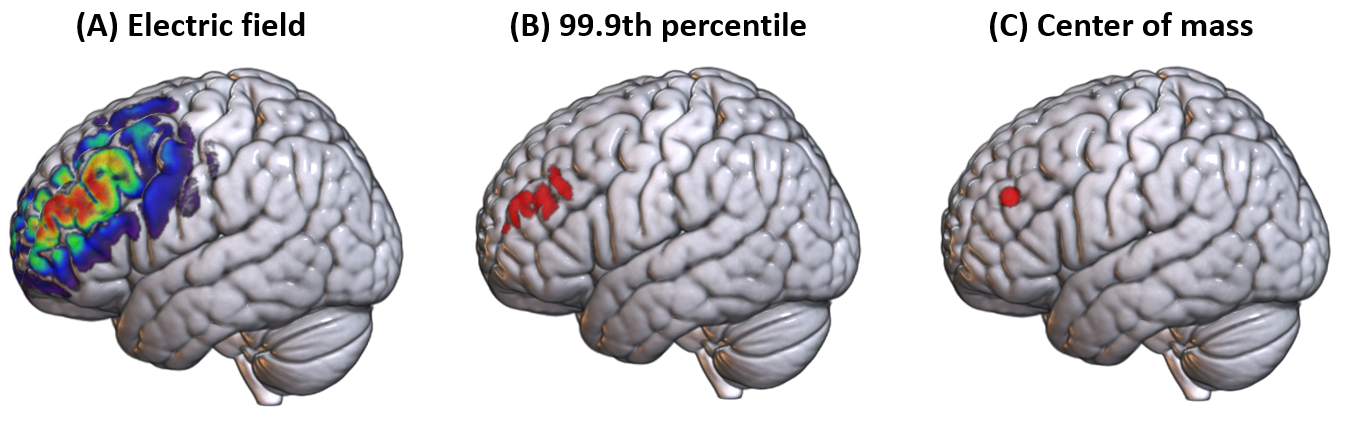
**

**Figure S1: Example for derivation of electric field center of mass.** To visualize the stimulated brain regions, the electric field was simulated for each participant based on actual coil position and orientation during the measurement. Subsequently, the 99.9th whole brain percentile was identified based on electric field magnitude. Finally, the center of mass for this area of maximum electric field was derived and used to visualize the stimulated area for each participant.

**Resting state preprocessing.** Preprocessing of resting state data was performed using an in-house developed pipeline (RStools). An exact stepwise process of the preprocessing and analysis can be found in the appendix. In summary, data was de-spiked (AFNI 3dDespike), slice-time corrected (FSL), bias-field corrected (ANTs) and realignment (FSL) was performed. Next, spatial normalization to MNI space (ANTs) and smoothing with a 6 mm FWHM Gaussian kernel (FSL) was performed. To calculate the desired resting state connectivity of the sgACC, we applied seed-voxel correlation using the sgACC as a seed as defined by Fox *et al.* [4].

***Supplementary* *results***


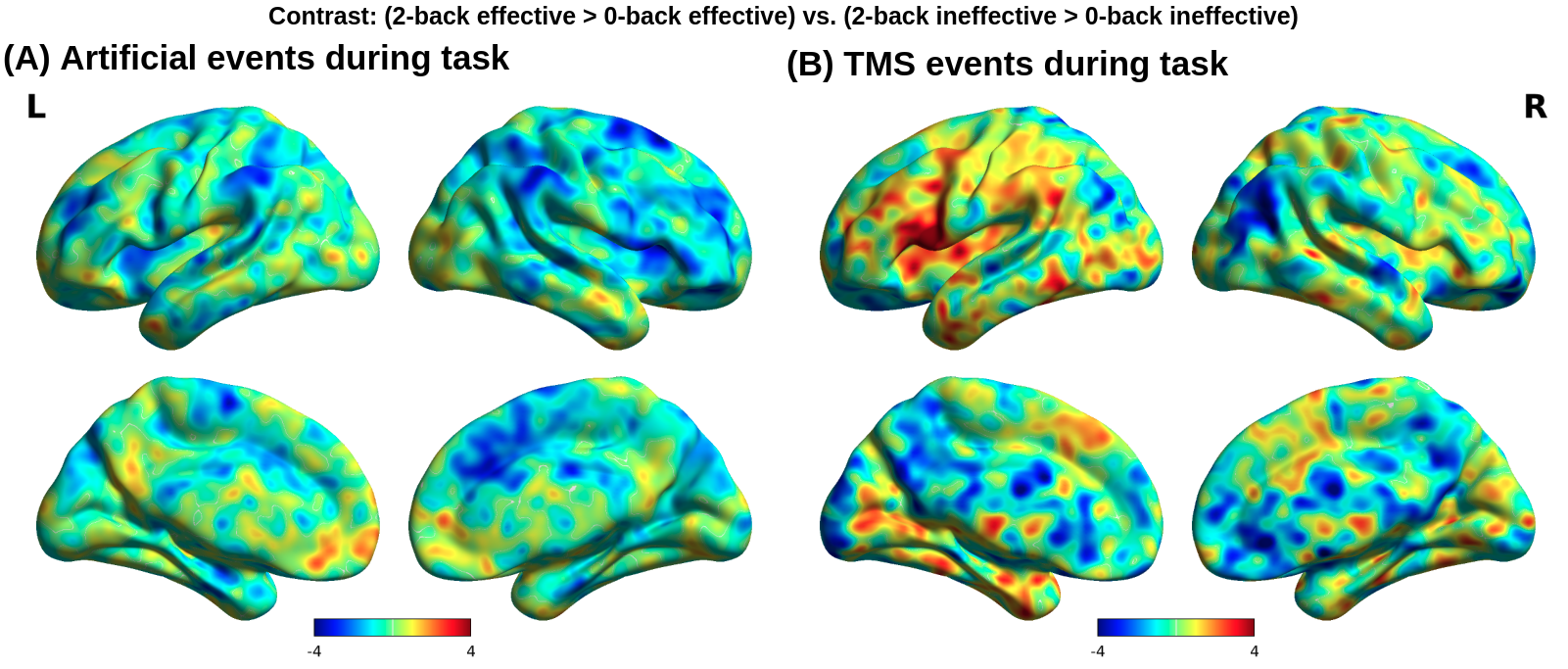


**Figure S2: Comparing unthresholded t-maps of the target contrast created using artificial events during the task compared to task with TMS.** In areas showing the significant activations as depicted in the manuscript (left IFG, right IPS, right sgACC), effects are way stronger or even reversed with TMS compared to task only. Voxelwise spatial correlation coefficient between maps is -0.14.


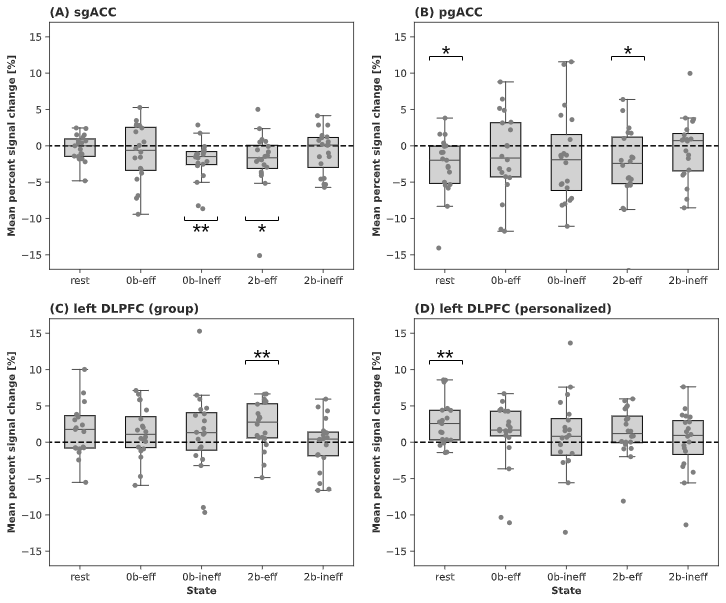


**Figure S3: Results for ROI analysis in terms of subject-wise mean percent signal change for each individual state in the predefined ROIs.** Statistically significant results (one sample t-test, testing against a population mean of 0) are marked with asterisks (* corresponds to p<0.05, ** indicates p<0.005).


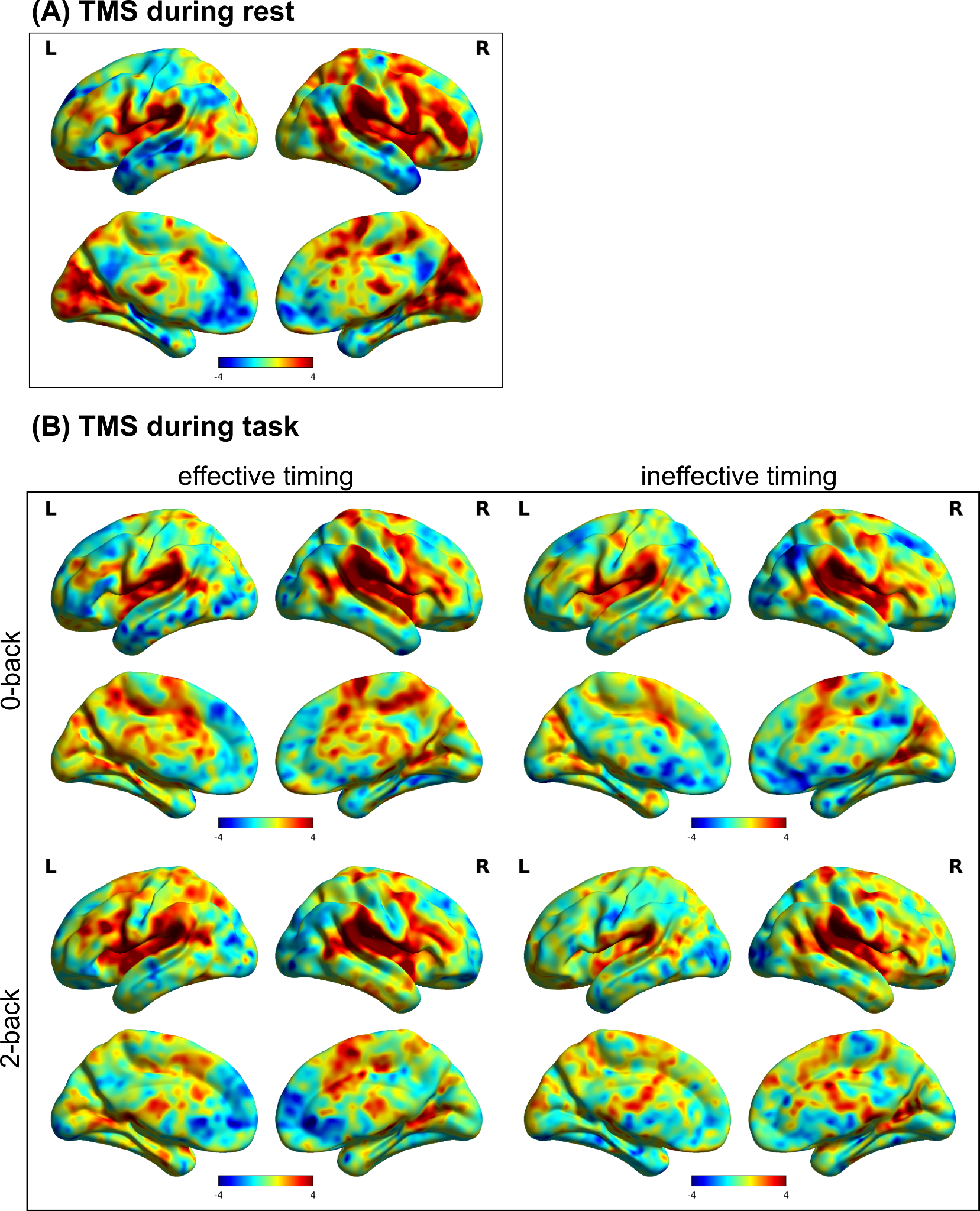


**Figure S4. Group level t-maps for TMS effects during rest (A) compared to chronometric TMS during N-back task (B).** Each map was derived using a one-sample t-test on subject-level parameter estimates derived from 16 triplets of TMS per condition. During rest, maps also show activations of visual areas which are not present for TMS during the task, as the basic task activation including visual areas is part of a separate regressor.


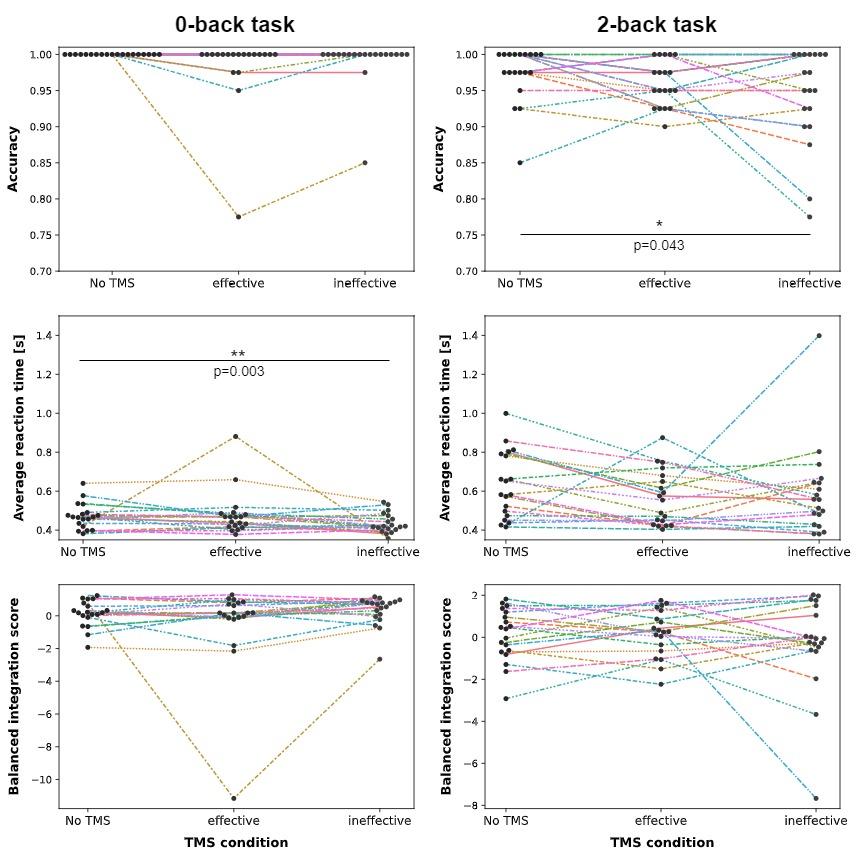


**Figure S5: Accuracy, average reaction time and balanced integration score [6] for 0-back and 2-back task with no TMS as well as effective and ineffective TMS timings.** Behavior was analyzed for 14 participants, lines connect corresponding data-points for each participant. Statistically significant differences are marked with an asterisk (paired t-test, p<0.05) and corresponding p-values are provided. Changes in behavior could be identified only for reaction time during 0-back task.

***
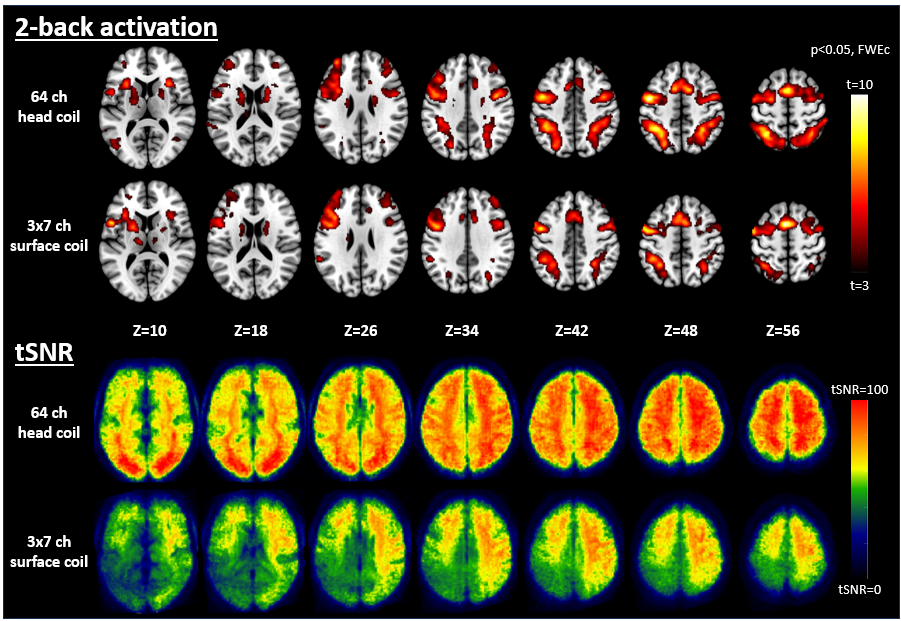
***

**Figure S6: Comparison of activation maps and temporal signal to noise ratio for a standard head-coil (64 channels) and the dedicated TMS-fMRI coils (3x7 channels).** Group level activation maps for the 2-back task (p<0.05, FWE corrected on cluster level, p<0.001 cluster identification threshold) are shown at the top part of the figure. We could not detect a statistically significant difference between activation maps. In terms of tSNR (bottom part), the 64-ch head coil results in more homogeneous whole-brain SNR and increased values especially in inferior brain regions, e.g. the occipital cortex.


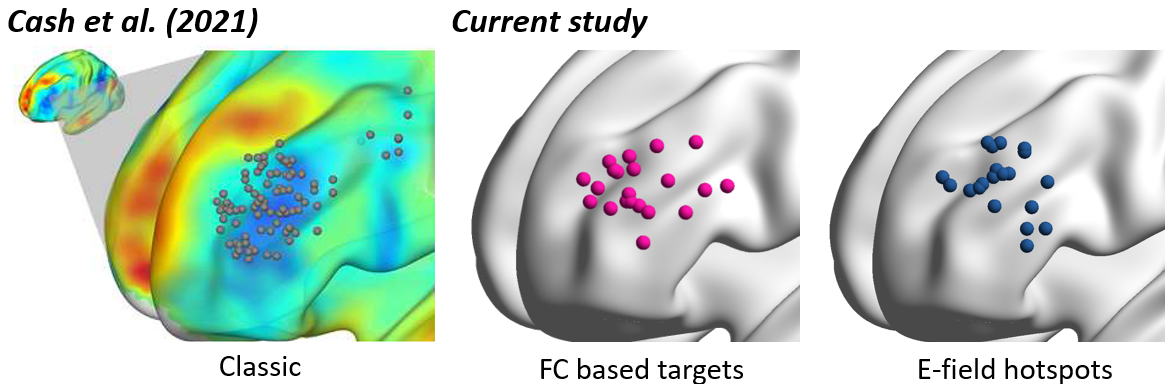


**Figure S7: Comparison of individualized targets between previously published results and the current study. Figures on the left were taken from Cash et al. (2021)** [5]**.** Individualized targets were defined based on functional connectivity. Subsequently, we also calculated E-field hotspots based on actual coil pose (position and orientation). Both clouds overlap with targets defined by Cash et al, strengthening the targeting approach used within this study.

***
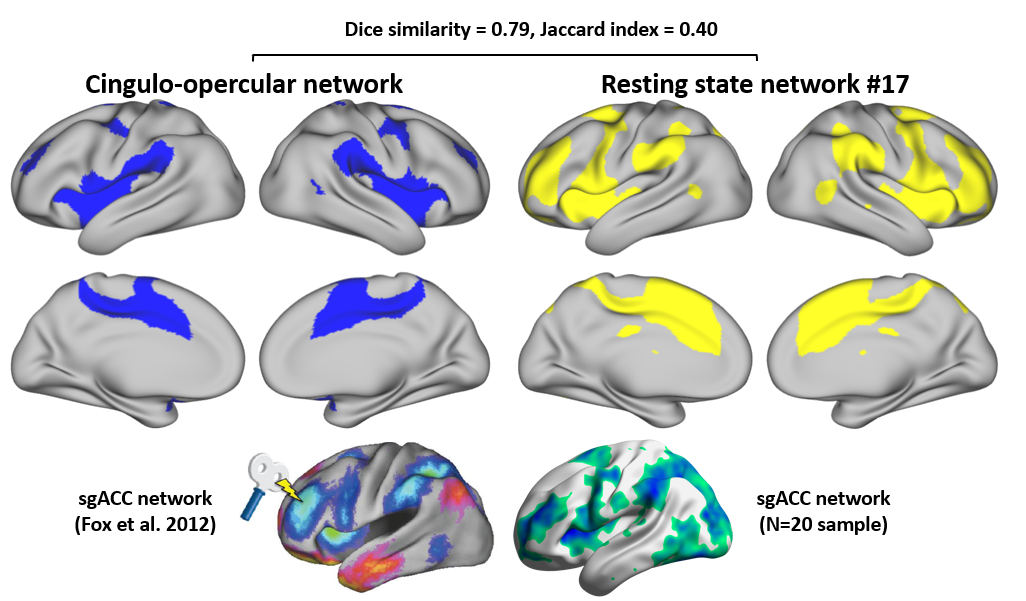
***

**Figure S8: Comparison of cingulo-opercular network and targeted resting state network #17.** While the cingulo-opercular network [7] has been associated with uncomfortable stimuli, it also shows large overlap and high similarity with the resting state network #17 [8], which has been shown to change functional connectivity after application of TMS [8]. Importantly, both networks also show high similarity to the sgACC network associated with higher TMS efficacy by Fox et al. [4] and the average sgACC network within this study.

***Supplementary Bibliography***

1. Thielscher A, Antunes A, Saturnino GB. Field modeling for transcranial magnetic stimulation: A useful tool to understand the physiological effects of TMS? 2015 37th Annual International Conference of the IEEE Engineering in Medicine and Biology Society (EMBC), 2015. p. 222–225.

2. Drakaki M, Mathiesen C, Siebner HR, Madsen K, Thielscher A. Database of 25 validated coil models for electric field simulations for TMS. Brain Stimul. 2022;15:697–706.

3. Puonti O, Van Leemput K, Saturnino GB, Siebner HR, Madsen KH, Thielscher A. Accurate and robust whole-head segmentation from magnetic resonance images for individualized head modeling. NeuroImage. 2020;219:117044.

4. Fox MD, Buckner RL, White MP, Greicius MD, Pascual-Leone A. Efficacy of transcranial magnetic stimulation targets for depression is related to intrinsic functional connectivity with the subgenual cingulate. Biol Psychiatry. 2012;72:595–603.

5. Cash RFH, Cocchi L, Lv J, Wu Y, Fitzgerald PB, Zalesky A. Personalized connectivity-guided DLPFC-TMS for depression: Advancing computational feasibility, precision and reproducibility. Hum Brain Mapp. 2021;42:4155–4172.

6. Liesefeld HR, Janczyk M. Combining speed and accuracy to control for speed-accuracy trade-offs(?). Behav Res. 2019;51:40–60.

7. Lynch CJ, Elbau I, Ng T, Ayaz A, Zhu S, Manfredi N, et al. Expansion of a frontostriatal salience network in individuals with depression. 2023:2023.08.09.551651.

8. Tik M, Hoffmann A, Sladky R, Tomova L, Hummer A, Navarro de Lara L, et al. Towards understanding rTMS mechanism of action: Stimulation of the DLPFC causes network-specific increase in functional connectivity. Neuroimage. 2017;162:289–296.

***Appendix: Resting state preprocessing pipeline***

Task #1 Reorient EPI to LPI

Tool: Orientation

Params:

--orientation=LPI

--output=/path/to/study/preproc/rs/subject/vols_LPI.nii

--input=/path/to/study/subjects/subject/M1/rs/nifti/vols.nii

--dicom=/path/to/study/subjects/subject/…/dicom

--phaseencdir=y+

Task #2 De-spiking

Tool: De-spiking

Params:

--output=/path/to/study/preproc/rs/subject/dvols.nii

--input=/path/to/study/preproc/rs/subject/vols_LPI.nii

Cmd:

rm -f /path/to/study/preproc/rs/subject/dvols.nii

/usr/lib/afni/bin/3dDespike -prefix /path/to/study/preproc/rs/subject/dvols.nii -nomask -q /path/to/study/preproc/rs/subject/vols_LPI.nii

Task #3 Slice Timing

Tool: Slice Timing

Params:

--in=/path/to/study/preproc/rs/subject/dvols.nii

--repeat=2

--mean=/path/to/study/preproc/rs/subject/meanadvols.nii

--out=/path/to/study/preproc/rs/subject/advols.nii

--odd

Cmd:

/usr/lib/fsl/5.0/slicetimer --in=/path/to/study/preproc/rs/subject/dvols.nii --out=/path/to/study/preproc/rs/subject/advols.nii --repeat=2 --direction=3 --odd

/usr/lib/fsl/5.0/fslmaths /path/to/study/preproc/rs/subject/advols.nii -Tmean /path/to/study/preproc/rs/subject/meanadvols.nii

Task #4 Bias Field Correction

Tool: Bias Field Correction

Params:

--shrinkfactor=2

--input=/path/to/study/preproc/rs/subject/advols.nii

--secondaryInput=/path/to/study/preproc/rs/subject/meanadvols.nii

--output=/path/to/study/preproc/rs/subject/badvols.nii

--bfield=/path/to/study/preproc/rs/subject/bf.nii

Cmd:

tmpdir=`mktemp -d 2>/dev/null || mktemp -d -t 'rstools-pps'`

/usr/lib/ants/N4BiasFieldCorrection -i /path/to/study/preproc/rs/subject/meanadvols.nii -d 3 -s 2 -o [$tmpdir/corrected.nii,/path/to/study/preproc/rs/subject/bf.nii]

/usr/lib/fsl/5.0/fslmaths /path/to/study/preproc/rs/subject/advols.nii -div /path/to/study/preproc/rs/subject/bf.nii /path/to/study/preproc/rs/subject/badvols.nii

rm -rf $tmpdir

Task #5 Realignment

Tool: Realignment

Params:

--stages=4

--input=/path/to/study/preproc/rs/subject/badvols.nii

--output=/path/to/study/preproc/rs/subject/rbadvols.nii

--mean=/path/to/study/preproc/rs/subject/meanrbadvols.nii

Cmd:

/usr/lib/fsl/5.0/mcflirt -in /path/to/study/preproc/rs/subject/badvols.nii -out /path/to/study/preproc/rs/subject/rbadvols.nii -stages 4 -mats -plots -meanvol

/usr/lib/fsl/5.0/fslmaths /path/to/study/preproc/rs/subject/rbadvols.nii -Tmean /path/to/study/preproc/rs/subject/meanrbadvols.nii

Task #6 Realignment Transformation

Tool: Unified Transformation

Params:

--input=/path/to/study/preproc/rs/subject/advols.nii

--output=/path/to/study/preproc/rs/subject/irbadvols.nii

--reference=/path/to/study/preproc/rs/subject/meanrbadvols.nii

--headerReference=/path/to/study/preproc/rs/subject/rbadvols.nii

--trans=div,/path/to/study/preproc/rs/subject/bf.nii

--trans=mcflirt,/path/to/study/preproc/rs/subject/rbadvols.nii.mat

--defaultValue=0.0

Task #7 Compute Template Registration

Tool: Normalization

Params:

--epiTemplate=/path/preprocessing/template/3t_epi_template_LPI.nii

--epi2EpiTemplateWarp=/path/to/study/preproc/rs/subject/epi2tpl_Warp.nii.gz

--epi2EpiTemplateInvWarp=/path/to/study/preproc/rs/subject/epi2tpl_InverseWarp.nii.gz

--epi2EpiTemplateAffine=/path/to/study/preproc/rs/subject/epi2tpl_affine.txt

--input=/path/to/study/preproc/rs/subject/irbadvols.nii

--mean=/path/to/study/preproc/rs/subject/meanirbadvols.nii

Cmd:

export ITK_GLOBAL_DEFAULT_NUMBER_OF_THREADS=1

# create a temporary directory

tmpdir=`mktemp -d 2>/dev/null || mktemp -d -t 'rstools-pps'`

echo "Temporary directory: "$tmpdir

# compute mean

/usr/lib/fsl/5.0/fslmaths /path/to/study/preproc/rs/subject/irbadvols.nii -Tmean /path/to/study/preproc/rs/subject/meanirbadvols.nii

# compute EPI template edges

/usr/lib/fsl/5.0/fslmaths /path/preprocessing/template/3t_epi_template_LPI.nii -kernel file /usr/local/share/rstools-preprocessing/utils/logkernel_0.3.nii.gz -fmeanu $tmpdir/edges_tpl.nii

# compute EPI edges

/usr/local/bin/rsdeoblique -i /path/to/study/preproc/rs/subject/meanirbadvols.nii -r $tmpdir/deob_trans.txt -o $tmpdir/deobliqued_input.nii -v

/usr/local/bin/rszeropadding -i $tmpdir/deobliqued_input.nii -o $tmpdir/padded_input.nii -a 5 -b 5 -c 5 -d 5 -e 5 -f 5 -v

/usr/lib/fsl/5.0/fslmaths $tmpdir/padded_input.nii -kernel file /usr/local/share/rstools-preprocessing/utils/logkernel_0.3.nii.gz -fmeanu $tmpdir/edges_input.nii

# register EPI edges to EPI template edges

/usr/lib/ants/antsRegistration --verbose 1 --dimensionality 3 --float 0 --output "[$tmpdir/tpl,$tmpdir/tplWarped.nii.gz]" --interpolation Linear --winsorize-image-intensities "[0.005,0.995]" --use-histogram-matching 0 --initial-moving-transform "[$tmpdir/edges_tpl.nii,$tmpdir/edges_input.nii,0]" --transform "Rigid[0.1]" --metric "MI[$tmpdir/edges_tpl.nii,$tmpdir/edges_input.nii,1,32,Regular,0.25]" --convergence "[1000x500x250x100,1e-6,10]" --shrink-factors 8x4x2x1 --smoothing-sigmas 3x2x1x0vox --transform "Affine[0.1]" --metric "MI[$tmpdir/edges_tpl.nii,$tmpdir/edges_input.nii,1,32,Regular,0.25]" --convergence "[1000x500x250x100,1e-6,10]" --shrink-factors 8x4x2x1 --smoothing-sigmas 3x2x1x0vox --transform "SyN[0.1,3,0]" --metric "CC[$tmpdir/edges_tpl.nii,$tmpdir/edges_input.nii,1,4]" --convergence "[100x70x50x20,1e-6,10]" --shrink-factors 8x4x2x1 --smoothing-sigmas 3x2x1x0vox

# warp brain mask

/usr/lib/ants/antsApplyTransforms -e 3 -d 3 -i /path/preprocessing/template/t1_template_MNI_brainmask.nii -o $tmpdir/brainmask_tpl.nii -r $tmpdir/edges_tpl.nii -t "[/path/preprocessing/template/3t_epi_template_affine.txt,1]" -t /path/preprocessing/template/3t_epi_template_InverseWarp.nii.gz

/usr/lib/ants/antsApplyTransforms -e 3 -d 3 -i /path/preprocessing/template/t1_template_MNI_brainmask.nii -o $tmpdir/brainmask_input.nii -r $tmpdir/deobliqued_input.nii -t "[$tmpdir/tpl0GenericAffine.mat,1]" $tmpdir/tpl1InverseWarp.nii.gz -t "[/path/preprocessing/template/3t_epi_template_affine.txt,1]" -t /path/preprocessing/template/3t_epi_template_InverseWarp.nii.gz

# create field of view mask

/usr/lib/fsl/5.0/fslmaths /path/to/study/preproc/rs/subject/meanirbadvols.nii -thr 1 -uthr 0 -add 1 $tmpdir/fov_input.nii

/usr/lib/ants/antsApplyTransforms -e 3 -d 3 -i $tmpdir/fov_input.nii -o $tmpdir/deobliqued_fov_input.nii -r $tmpdir/deobliqued_input.nii

/usr/lib/fsl/5.0/fslmaths $tmpdir/brainmask_input.nii -mas $tmpdir/deobliqued_fov_input.nii $tmpdir/fov_brainmask_input.nii

# create epi template mask

/usr/lib/fsl/5.0/fslmaths /path/preprocessing/template/3t_epi_template_LPI.nii -mas $tmpdir/brainmask_tpl.nii $tmpdir/brainmasked_tpl.nii

csfValue=$(/usr/lib/fsl/5.0/fslstats $tmpdir/brainmasked_tpl.nii -k $tmpdir/brainmask_tpl.nii -p 95)

/usr/lib/fsl/5.0/fslmaths $tmpdir/brainmask_tpl.nii -binv -mul $csfValue $tmpdir/brainmask_inv_tpl.nii

/usr/lib/fsl/5.0/fslmaths $tmpdir/brainmasked_tpl.nii -add $tmpdir/brainmask_inv_tpl.nii $tmpdir/skullstripped_tpl.nii

# create epi mask

/usr/lib/fsl/5.0/fslmaths $tmpdir/deobliqued_input.nii -mas $tmpdir/fov_brainmask_input.nii $tmpdir/brainmasked_input.nii

csfValue=$(/usr/lib/fsl/5.0/fslstats $tmpdir/brainmasked_input.nii -k $tmpdir/fov_brainmask_input.nii -p 95)

/usr/lib/fsl/5.0/fslmaths $tmpdir/fov_brainmask_input.nii -binv -mul $csfValue $tmpdir/brainmask_inv_input.nii

/usr/lib/fsl/5.0/fslmaths $tmpdir/brainmasked_input.nii -add $tmpdir/brainmask_inv_input.nii $tmpdir/skullstripped_input.nii

# perform registration

## get the rigid part

/usr/lib/ants/antsRegistration --verbose 1 --dimensionality 3 --float 0 --output "[$tmpdir/final_tpl,$tmpdir/final_tplWarped.nii.gz]" --interpolation Linear --winsorize-image-intensities "[0.005,0.995]" --use-histogram-matching 0 --initial-moving-transform "[$tmpdir/skullstripped_tpl.nii,$tmpdir/skullstripped_input.nii,1]" --transform "Rigid[0.1]" --metric "MI[$tmpdir/skullstripped_tpl.nii,$tmpdir/skullstripped_input.nii,1,32,Regular,0.25]" --convergence "[1000x500x250x100,1e-6,10]" --shrink-factors 8x4x2x1 --smoothing-sigmas 3x2x1x0vox --transform "Affine[0.1]" --metric "MI[$tmpdir/skullstripped_tpl.nii,$tmpdir/skullstripped_input.nii,1,32,Regular,0.25]" --convergence "[1000x500x250x100,1e-6,10]" --shrink-factors 8x4x2x1 --smoothing-sigmas 3x2x1x0vox

## get the deformable part

/usr/lib/ants/antsRegistration --verbose 1 -d 3 --initial-moving-transform $tmpdir/final_tpl0GenericAffine.mat -m "cc[$tmpdir/skullstripped_tpl.nii,$tmpdir/skullstripped_input.nii,1,2]" -t 'SyN[0.25,3,0]' -c 100x70x50x20 -f 8x4x2x1 -s 3x2x1x0 -o $tmpdir/final_tpl2

/usr/lib/ants/ConvertTransformFile 3 $tmpdir/final_tpl20GenericAffine.mat $tmpdir/final_tpl20GenericAffine.txt

# save resulting transformation parameters

cp $tmpdir/final_tpl21Warp.nii.gz /path/to/study/preproc/rs/subject/epi2tpl_Warp.nii.gz

cp $tmpdir/final_tpl21InverseWarp.nii.gz /path/to/study/preproc/rs/subject/epi2tpl_InverseWarp.nii.gz

cp $tmpdir/final_tpl20GenericAffine.txt /path/to/study/preproc/rs/subject/epi2tpl_affine.txt

rm -rf $tmpdir

Task #8 Transform EPI to MNI

Tool: Unified Transformation

Params:

--input=/path/to/study/preproc/rs/subject/advols.nii

--output=/path/to/study/preproc/rs/subject/iwrbadvols.nii

--reference=/path/preprocessing/template/t1_template_MNI_epi_space.nii

--headerReference=/path/to/study/preproc/rs/subject/meanirbadvols.nii

--trans=div,/path/to/study/preproc/rs/subject/bf.nii

--trans=mcflirt,/path/to/study/preproc/rs/subject/rbadvols.nii.mat

--trans=ants,/path/to/study/preproc/rs/subject/epi2tpl_affine.txt

--trans=ants,/path/to/study/preproc/rs/subject/epi2tpl_Warp.nii.gz

--trans=ants,/path/preprocessing/template/3t_epi_template_affine.txt

--trans=ants,/path/preprocessing/template/3t_epi_template_Warp.nii.gz

--space=mni

--defaultValue=0.0

Task #9 Transform mean EPI to MNI

Tool: Transformation

Params:

--input=/path/to/study/preproc/rs/subject/meanirbadvols.nii

--output=/path/to/study/preproc/rs/subject/wmeanrbadvols_mnitpl.nii

--reference=/path/preprocessing/template/t1_template_MNI.nii

--transformationType=epi2mni

--epi2EpiTemplateWarp=/path/to/study/preproc/rs/subject/epi2tpl_Warp.nii.gz

--epi2EpiTemplateInvWarp=/path/to/study/preproc/rs/subject/epi2tpl_InverseWarp.nii.gz

--epi2EpiTemplateAffine=/path/to/study/preproc/rs/subject/epi2tpl_affine.txt

--epiTemplateWarp=/path/preprocessing/template/3t_epi_template_Warp.nii.gz

--epiTemplateInvWarp=/path/preprocessing/template/3t_epi_template_InverseWarp.nii.gz

--epiTemplateAffine=/path/preprocessing/template/3t_epi_template_affine.txt

--new

Cmd:

/usr/lib/ants/antsApplyTransforms -e 3 -d 3 -i /path/to/study/preproc/rs/subject/meanirbadvols.nii -o /path/to/study/preproc/rs/subject/wmeanrbadvols_mnitpl.nii -r /path/preprocessing/template/t1_template_MNI.nii -t /path/preprocessing/template/3t_epi_template_Warp.nii.gz -t /path/preprocessing/template/3t_epi_template_affine.txt -t /path/to/study/preproc/rs/subject/epi2tpl_Warp.nii.gz -t /path/to/study/preproc/rs/subject/epi2tpl_affine.txt

Task #10 Transform mean EPI to EPI-template space

Tool: Transformation

Params:

--input=/path/to/study/preproc/rs/subject/meanirbadvols.nii

--output=/path/to/study/preproc/rs/subject/wmeanrbadvols_epitpl.nii

--reference=/path/preprocessing/template/3t_epi_template_LPI.nii

--transformationType=epi2epitpl

--epi2EpiTemplateWarp=/path/to/study/preproc/rs/subject/epi2tpl_Warp.nii.gz

--epi2EpiTemplateInvWarp=/path/to/study/preproc/rs/subject/epi2tpl_InverseWarp.nii.gz

--epi2EpiTemplateAffine=/path/to/study/preproc/rs/subject/epi2tpl_affine.txt

--epiTemplateWarp=/path/preprocessing/template/3t_epi_template_Warp.nii.gz

--epiTemplateInvWarp=/path/preprocessing/template/3t_epi_template_InverseWarp.nii.gz

--epiTemplateAffine=/path/preprocessing/template/3t_epi_template_affine.txt

--new

Cmd:

/usr/lib/ants/antsApplyTransforms -e 3 -d 3 -i /path/to/study/preproc/rs/subject/meanirbadvols.nii -o /path/to/study/preproc/rs/subject/wmeanrbadvols_epitpl.nii -r /path/preprocessing/template/3t_epi_template_LPI.nii -t /path/to/study/preproc/rs/subject/epi2tpl_Warp.nii.gz -t /path/to/study/preproc/rs/subject/epi2tpl_affine.txt

Task #11 Transform EPI-template into EPI space

Tool: Transformation

Params:

--input=/path/preprocessing/template/3t_epi_template_LPI.nii

--output=/path/to/study/preproc/rs/subject/epitpl_epi_space.nii

--reference=/path/to/study/preproc/rs/subject/meanirbadvols.nii

--transformationType=epitpl2epi

--epi2EpiTemplateWarp=/path/to/study/preproc/rs/subject/epi2tpl_Warp.nii.gz

--epi2EpiTemplateInvWarp=/path/to/study/preproc/rs/subject/epi2tpl_InverseWarp.nii.gz

--epi2EpiTemplateAffine=/path/to/study/preproc/rs/subject/epi2tpl_affine.txt

--epiTemplateWarp=/path/preprocessing/template/3t_epi_template_Warp.nii.gz

--epiTemplateInvWarp=/path/preprocessing/template/3t_epi_template_InverseWarp.nii.gz

--epiTemplateAffine=/path/preprocessing/template/3t_epi_template_affine.txt

--new

Cmd:

/usr/lib/ants/antsApplyTransforms -e 3 -d 3 -i /path/preprocessing/template/3t_epi_template_LPI.nii -o /path/to/study/preproc/rs/subject/epitpl_epi_space.nii -r /path/to/study/preproc/rs/subject/meanirbadvols.nii -t "[/path/to/study/preproc/rs/subject/epi2tpl_affine.txt,1]" -t /path/to/study/preproc/rs/subject/epi2tpl_InverseWarp.nii.gz

Task #12 Transform T1 MNI-template into EPI space

Tool: Transformation

Params:

--input=/path/preprocessing/template/t1_template_MNI.nii

--output=/path/to/study/preproc/rs/subject/t1_epi_space_MNI.nii

--reference=/path/to/study/preproc/rs/subject/meanirbadvols.nii

--transformationType=mni2epi

--epi2EpiTemplateWarp=/path/to/study/preproc/rs/subject/epi2tpl_Warp.nii.gz

--epi2EpiTemplateInvWarp=/path/to/study/preproc/rs/subject/epi2tpl_InverseWarp.nii.gz

--epi2EpiTemplateAffine=/path/to/study/preproc/rs/subject/epi2tpl_affine.txt

--epiTemplateWarp=/path/preprocessing/template/3t_epi_template_Warp.nii.gz

--epiTemplateInvWarp=/path/preprocessing/template/3t_epi_template_InverseWarp.nii.gz

--epiTemplateAffine=/path/preprocessing/template/3t_epi_template_affine.txt

--new

Cmd:

/usr/lib/ants/antsApplyTransforms -e 3 -d 3 -i /path/preprocessing/template/t1_template_MNI.nii -o /path/to/study/preproc/rs/subject/t1_epi_space_MNI.nii -r /path/to/study/preproc/rs/subject/meanirbadvols.nii -t "[/path/to/study/preproc/rs/subject/epi2tpl_affine.txt,1]" -t /path/to/study/preproc/rs/subject/epi2tpl_InverseWarp.nii.gz -t "[/path/preprocessing/template/3t_epi_template_affine.txt,1]" -t /path/preprocessing/template/3t_epi_template_InverseWarp.nii.gz

Task #13 Transform MNI-brainmask into EPI space

Tool: Transformation

Params:

--input=/path/preprocessing/template/t1_template_MNI_brainmask.nii

--output=/path/to/study/preproc/rs/subject/brainmask.nii

--reference=/path/to/study/preproc/rs/subject/meanirbadvols.nii

--transformationType=mni2epi

--epi2EpiTemplateWarp=/path/to/study/preproc/rs/subject/epi2tpl_Warp.nii.gz

--epi2EpiTemplateInvWarp=/path/to/study/preproc/rs/subject/epi2tpl_InverseWarp.nii.gz

--epi2EpiTemplateAffine=/path/to/study/preproc/rs/subject/epi2tpl_affine.txt

--epiTemplateWarp=/path/preprocessing/template/3t_epi_template_Warp.nii.gz

--epiTemplateInvWarp=/path/preprocessing/template/3t_epi_template_InverseWarp.nii.gz

--epiTemplateAffine=/path/preprocessing/template/3t_epi_template_affine.txt

--new

Cmd:

/usr/lib/ants/antsApplyTransforms -e 3 -d 3 -i /path/preprocessing/template/t1_template_MNI_brainmask.nii -o /path/to/study/preproc/rs/subject/brainmask.nii -r /path/to/study/preproc/rs/subject/meanirbadvols.nii -t "[/path/to/study/preproc/rs/subject/epi2tpl_affine.txt,1]" -t /path/to/study/preproc/rs/subject/epi2tpl_InverseWarp.nii.gz -t "[/path/preprocessing/template/3t_epi_template_affine.txt,1]" -t /path/preprocessing/template/3t_epi_template_InverseWarp.nii.gz

Task #14 Compute volumes with severe motion artifacts

Tool: Motion Scrubbing

Params:

--input=/path/to/study/preproc/rs/subject/vols_LPI.nii

--rp=/path/to/study/preproc/rs/subject/rbadvols.nii.par.txt

--mask=/path/to/study/preproc/rs/subject/brainmask.nii

--dvars=/path/to/study/preproc/rs/subject/ms_dvars.txt

--fd=/path/to/study/preproc/rs/subject/ms_fd.txt

--flagged=/path/to/study/preproc/rs/subject/ms_flagged.txt

--dvarsthreshold=0.04

--fdthreshold=0.6

--rpformat=fsl

Task #15 Smoothing

Tool: Smoothing

Params:

--kernelSize=6.0

--input=/path/to/study/preproc/rs/subject/iwrbadvols.nii

--output=/path/to/study/preproc/rs/subject/siwrbadvols.nii

Task #16 Extracting the global mean signal

Tool: Extract Timecourse

Params:

--mask=/path/preprocessing/template/t1_template_MNI_brainmask.nii

--input=/path/to/study/preproc/rs/subject/iwrbadvols.nii

--output=/path/to/study/preproc/rs/subject/tc_global_iwrbadvols.txt

Task #17 Extracting the mean CSF signal

Tool:Extract Timecourse

Params:

--mask=/path/preprocessing/masks/roi_csf.nii

--input=/path/to/study/preproc/rs/subject/iwrbadvols.nii

--output=/path/to/study/preproc/rs/subject/mean_tc_csf_iwrbadvols.txt

Task #18 Extracting the mean WM signal

Tool: Extract Timecourse

Params:

--mask=/path/preprocessing/masks/roi_wm.nii

--input=/path/to/study/preproc/rs/subject/iwrbadvols.nii

--output=/path/to/study/preproc/rs/subject/mean_tc_iwm_wrbadvols.txt

Task #19 Extracting the first 5 PCA-components of the CSF signal

Tool: Extract Timecourse

Params:

--mask=/path/preprocessing/masks/roi_csf.nii

--input=/path/to/study/preproc/rs/subject/iwrbadvols.nii

--output=/path/to/study/preproc/rs/subject/tc_csf_iwrbadvols.txt

--algorithm=tpca

--retainComponents=5

--useStandardScores

Task #20 Extracting the first 5 PCA-components of the WM signal

Tool: Extract Timecourse

Params:

--mask=/path/preprocessing/masks/roi_wm.nii

--input=/path/to/study/preproc/rs/subject/iwrbadvols.nii

--output=/path/to/study/preproc/rs/subject/tc_wm_iwrbadvols.txt

--algorithm=tpca

--retainComponents=5

--useStandardScores

Task #21 Merging all regressors into one file

Tool: Merge Regressors

Params:

--input=/path/to/study/preproc/rs/subject/tc_global_iwrbadvols.txt

--input=/path/to/study/preproc/rs/subject/mean_tc_csf_iwrbadvols.txt

--input=/path/to/study/preproc/rs/subject/tc_csf_iwrbadvols.txt

--input=/path/to/study/preproc/rs/subject/mean_tc_iwm_wrbadvols.txt

--input=/path/to/study/preproc/rs/subject/tc_wm_iwrbadvols.txt

--input=/path/to/study/preproc/rs/subject/rbadvols.nii.par.txt

--output=/path/to/study/preproc/rs/subject/tc_wm_csf_global_rp_iwrbadvols.txt

Cmd:

paste -d ' ' /path/to/study/preproc/rs/subject/tc_global_iwrbadvols.txt /path/to/study/preproc/rs/subject/mean_tc_csf_iwrbadvols.txt /path/to/study/preproc/rs/subject/tc_csf_iwrbadvols.txt /path/to/study/preproc/rs/subject/mean_tc_iwm_wrbadvols.txt /path/to/study/preproc/rs/subject/tc_wm_iwrbadvols.txt /path/to/study/preproc/rs/subject/rbadvols.nii.par.txt | sed -e 's/ / /g' | sed -e 's/ / /g' | sed -e 's/ / /g' > /path/to/study/preproc/rs/subject/tc_wm_csf_global_rp_iwrbadvols.txt

Task #22 Merging all regressors (excl. global mean) into one file

Tool: Merge Regressors

Params:

--input=/path/to/study/preproc/rs/subject/mean_tc_csf_iwrbadvols.txt

--input=/path/to/study/preproc/rs/subject/tc_csf_iwrbadvols.txt

--input=/path/to/study/preproc/rs/subject/mean_tc_iwm_wrbadvols.txt

--input=/path/to/study/preproc/rs/subject/tc_wm_iwrbadvols.txt

--input=/path/to/study/preproc/rs/subject/rbadvols.nii.par.txt

--output=/path/to/study/preproc/rs/subject/tc_wm_csf_rp_iwrbadvols.txt

Cmd:

paste -d ' ' /path/to/study/preproc/rs/subject/mean_tc_csf_iwrbadvols.txt /path/to/study/preproc/rs/subject/tc_csf_iwrbadvols.txt /path/to/study/preproc/rs/subject/mean_tc_iwm_wrbadvols.txt /path/to/study/preproc/rs/subject/tc_wm_iwrbadvols.txt /path/to/study/preproc/rs/subject/rbadvols.nii.par.txt | sed -e 's/ / /g' | sed -e 's/ / /g' | sed -e 's/ / /g' > /path/to/study/preproc/rs/subject/tc_wm_csf_rp_iwrbadvols.txt

Task #23 Regressing out nuisance signals

Tool: Regression

Params:

--input=/path/to/study/preproc/rs/subject/siwrbadvols.nii

--residuals=/path/to/study/preproc/rs/subject/ResI_siwrbadvols.nii

--regressors=/path/to/study/preproc/rs/subject/tc_wm_csf_global_rp_iwrbadvols.txt

Task #24 Applying the bandpass filter

Tool: Bandpass Filter

Params:

--f1=0.009

--f2=0.08

--input=/path/to/study/preproc/rs/subject/ResI_siwrbadvols.nii

--filtered=/path/to/study/preproc/rs/subject/Filtered_ResI_siwrbadvols.nii

--TR=2

Task #25 Remove volumes with severe motion

Tool: Apply Scrubbing

Params:

--input=/path/to/study/preproc/rs/subject/Filtered_ResI_siwrbadvols.nii

--output=/path/to/study/preproc/rs/subject/Scrubbed_Filtered_ResI_siwrbadvols.nii

--flagged=/path/to/study/preproc/rs/subject/ms_flagged.txt

Task #26 Extracting the mean signal in the ROI seed region

Tool: Extract Timecourse

Params:

--mask=/path/preprocessing/masks/mask.nii

--input=/path/to/study/preproc/rs/subject/Scrubbed_Filtered_ResI_siwrbadvols.nii

--output=/path/to/study/preproc/rs/subject/tc_mask.txt

Task #27 Seed voxel correlation analysis with the seed mask

Tool: Correlation

Params:

--input=/path/to/study/preproc/rs/subject/Scrubbed_Filtered_ResI_siwrbadvols.nii

--output=/path/to/study/preproc/rs/subject/fc_mask.nii

--regressor=/path/to/study/preproc/rs/subject/tc_mask.txt

--conversion=z
